# Complete Genome Sequence of the Deep-Sea Bacterium Moritella marina MP-1 (ATCC 15381)

**DOI:** 10.1128/MRA.01321-19

**Published:** 2020-01-23

**Authors:** Simon Magin, Anastasios Georgoulis, Konstantinos Papadimitriou, George Iliakis, Constantinos E. Vorgias

**Affiliations:** a Institute of Medical Radiation Biology, University of Duisburg-Essen, Medical School, Essen, Germany; b Department of Biochemistry and Molecular Biology, National and Kapodistrian University of Athens, Athens, Greece; c Department of Food Science and Technology, University of Peloponnese, Antikalamos, Kalamata, Greece; University of Southern California

## Abstract

Here, the complete assembly of the Moritella marina MP-1 (ATCC 15381) genome, combining Illumina and long Nanopore reads, is presented. The gapless assembly consists of a 4.7-Mb circular chromosome and a 26-kb plasmid, with a G+C content of 40.7%, and will assist in further studies of the molecular pathways in this biotechnologically significant organism.

## ANNOUNCEMENT

There is a high demand for polyunsaturated fatty acids (PUFAs) as a food supplement (1, 2). The marine bacterium Moritella marina (MP-1) has been reported to produce unusually high levels of the PUFA docosahexaenoic acid (DHA) (3, 4). This makes MP-1 interesting with regard to the biotechnological production of DHA (5). Currently, only 2 draft genome assemblies for MP-1 are publicly available (GenBank accession numbers GCA_000291685.1 and GCA_000381865.1). To address this, we used a hybrid assembly approach to generate a complete genome.

MP-1 was obtained from ATCC and grown in marine broth medium 2216 (catalog number BD279110) at 18°C for 72 hours in a conical flask agitated at 150 rpm. Two DNA preparations were purified from cultured bacteria using the NucleoSpin tissue kit (MachereyNagel), with one being subjected to paired-end sequencing on the HiSeq 2500 Illumina platform (MR DNA, TX) and the other used for Nanopore sequencing (Oxford Nanopore Technologies [ONT]) on a MinION instrument in our lab. The Illumina library was prepared using the Nextera DNA sample preparation kit (Illumina) with 41-ng input DNA, following the manufacturer’s user guide, which yielded an average library size of 1,370 bp, as determined with a 2100 bioanalyzer (Agilent Technologies). Library preparation for Nanopore sequencing was performed with the ONT rapid sequencing kit (catalog number SQK-RAD004) using 832-ng DNA as the input. A single sequencing run on a SpotON flow cell (R9.4.1) yielded 2.93 × 10^6^ reads, with a total of 8.99 × 10^9^ bases that were called with Albacore v2.2.6. Default parameters were used for all software unless specified otherwise. By filtering for reads longer than 20 kb and quality scores (Q) higher than 10 using the BBmap tool suite (v37.90) (6), 5,407 reads with a total of 1.31 × 10^8^ bases, corresponding to ∼28× coverage, were selected as input for the Canu assembly pipeline (v1.7) (7) set to an estimated genome size of 4.7 × 10^6^ bp. This resulted in assembly A of a gapless circular contig with a length of 4,733,441 bp. The use of a data set including shorter reads (>8 kb and Q > 10) from the same MinION run for assembly B with Canu at the same settings revealed a 26-kb circular extrachromosomal element. Analysis with PPR-Meta (1.0) identified the large contig as a bacterial chromosome and the 26-kb contig as a plasmid (8).

The chromosome assembled in A and the plasmid from B were used for downstream processing for sequence improvement. The increase in sequence accuracy was evaluated by comparison to the two available draft assemblies using MUMmer (9, 10). We employed signal-level analysis using NanoPolish (11), which increased the average alignment identity from 99.33% to 99.80%. (12). Using the previously generated Illumina data set (12.8 × 10^6^ reads, 250-bp length, ∼624× genome coverage), we ran 4 iterations of Pilon (v1.23), leading to final alignment identities of 99.98% to both draft assemblies. Duplicate overhangs were removed manually, resulting in final polished sequences of a 4,734,363-bp chromosome and a 26,062-bp plasmid.

Annotation with PGAP upon submission to NCBI yielded a total of 4,278 genes, including 198 RNA genes, comprised of 141 tRNA, 53 rRNA, and 4 noncoding RNA (ncRNA) genes (13). We expect that the availability of the complete genome sequence of MP-1 will pave the way for a more comprehensive study of this biotechnologically significant organism.

### Data availability.

The complete genome sequence has been deposited in GenBank under the accession number GCA_008931805 (CP044398 [plasmid] and CP044399 [chromosome]). The version described in this paper is the first version, GCA_008931805.1. Unfiltered raw sequencing reads have been deposited in the NCBI Sequence Read Archive under the accession numbers SRX6654081 (Nanopore) and SRX6827227 (Illumina).
